# Learning from cases: Analysis of two cases of craniopharyngioma from the 19 ^th^ to the 21 ^st^ centuries.

**DOI:** 10.12688/f1000research.19626.1

**Published:** 2019-08-30

**Authors:** John R. Apps, J. Ciaran Hutchinson, Susan Shelmerdine, Alex Virasami, Eduard Winter, Thomas S. Jacques, Juan-Pedro Martinez-Barbera, Owen Arthurs, Thomas Czech

**Affiliations:** 1 UCL Great Ormond Street Institute of Child Health, UCL, London, UK; 2 Cancer Research UK Clinical Trials Unit, University of Birmingham, UK, Birmingham, UK, UK; 3 Great Ormond Street Hospital for Children NHS Foundation Trust, London, UK; 4 Natural History Museum, Vienna, Austria; 5 Medical University Vienna, Vienna, Austria

**Keywords:** Craniopharyngiooma, historical, Erdheim, Engel, micro-CT

## Abstract

This manuscript describes the study of two cases of craniopharyngioma, which have been examined repeatedly over three separate centuries. This includes analysis by Josef Engel in 1839, who sought to uncover the physiological role of the pituitary gland; Jacob Erdheim in 1904, who initially described the disease we now call craniopharyngioma, and recent high resolution MRI and micro-CT imaging and attempted DNA analyses of the tumours. The cases highlight how, rightly or wrongly, our interpretation of data is shaped by the technologies, methodologies and prevailing theories of a given time.

The acquisition of knowledge from the study of individual cases is a core component of medical curricula across the world; it has been used to learn about and impart knowledge of human physiology, symptomatology, pathology, and clinical therapy for millennia ^1^ (McLean 2016). Combining cases into series has enabled the definition of diseases, define their natural history and forms an initial level of evidence in the evaluation of therapy ^2^ (Sayre, Toklu *et al*. 2017). Here, we present two cases of craniopharyngioma, where separate studies over three centuries have impacted on our understanding of the normal function of the pituitary gland and the classification of its pathology.

1828, in the seat of the Austrian Empire, a 33 year old Viennese waiter (Case 1) has been admitted to hospital complaining of longstanding weakness of his arms and legs, an increased need for sleep, headaches, intermittent vomiting, and a progressive deterioration of vision finally leading to complete blindness. Dying in a state of severe emaciation, post mortem examination identified a large cystic lesion in the region of the pituitary which was subsequently retained for further studies.

This patients tumour was kept, and next studied by Josef Engel, a pathologist completing his PhD thesis, “Über den Hirnanhang und den Trichter“ ( *On the pituitary and the infundibulum*) in 1839, working under the guidance of Carl von Rokitansky, one of the fathers of modern scientific pathology. At this time, the function of the pituitary gland was unknown. Through the characterisation of 12 cases of pituitary pathology, Engel proposed that the pituitary was a primitive version of the “small brain”, now known as the cerebellum. Remarkably, he postulated that the function of the cerebellum was for walking forwards, whereas the pituitary was for walking backwards. A drawing of the sample from the thesis is shown in Figure 1a.

**Figure 1.**
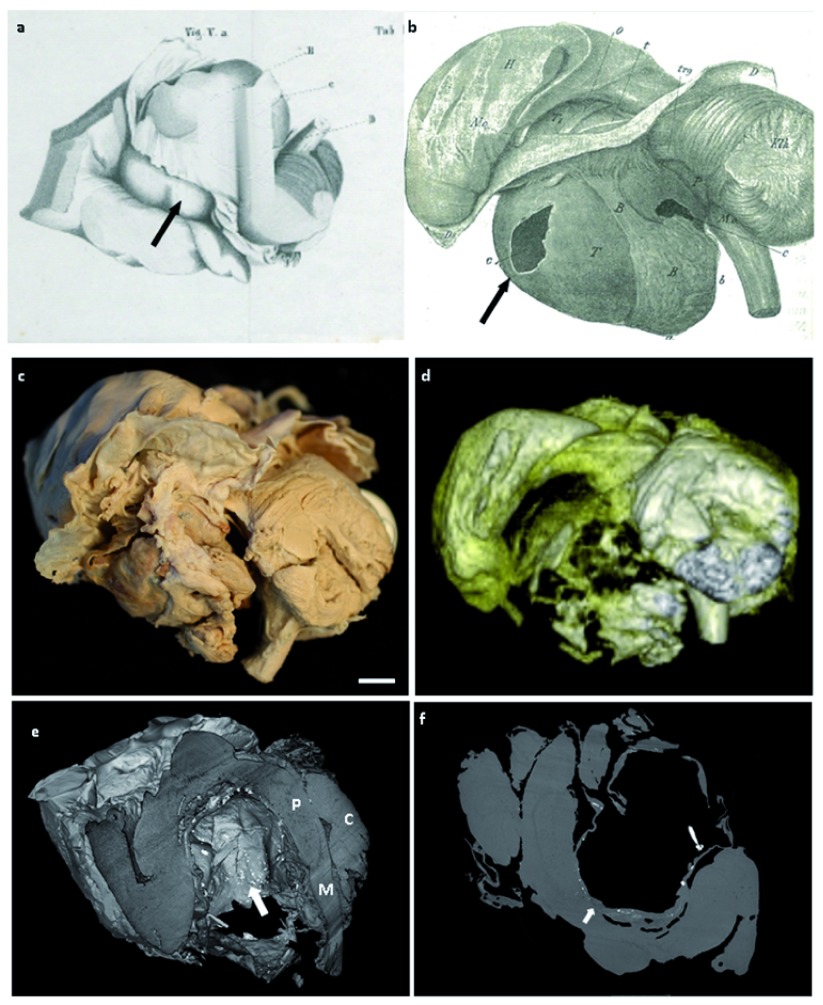
Case 1: Pathological drawing from **a**) Engel’s thesis in 1839 (Über den Hirnanhang und den Trichter, Medical University Vienna), **b**) Erdheim’s 1904 paper ^3^, showing a large infra-tentorial cystic lesion (arrow). **c**) Macroscopic photograph of specimen taken at Great Ormond Street Hospital. scale bar=1cm. **d**) 3T MRI reconstruction of the tumour matching the macroscopic figure. **e**) Micro-CT image showing cut-away of the cystic tumour with speckled hyper-intense areas consistent with calcification. P= Pons, M=Medulla, C=Cerebellum. **f**) Cross-sectional non-contrast micro-CT image showing cyst wall continuous with surrounding structures (arrow).

Whist this seems remarkable today, these conclusions were based on his careful observations. Engel noted several morphological correspondences between the pituitary gland and the cerebellum: both are covered by a tent of dura mater with an opposite semi-circular opening to connect with other parts of the brain, both lie within a bony depression in the midline skull base, both are “kidney-shaped”, both are bordered by a venous ring, the pituitary is surrounded by the arterial circle of Willis, while branches of the vertebral arteries enclose the cerebellum. Thus in some way he considered the pituitary gland a simplified replica of the cerebellum, much as others had considered the relationship between the cerebral hemispheres and the cerebellum. At the same time he also noted important differences; while the pituitary stalk slopes down in an anterior direction towards the gland, the cerebellar peduncle enters the cerebellum in an anterior-posterior direction.

Under the assumption of analogous form suggesting analogous function, observations regarding the influence of disease states of these structures on movement could be presented in a logical way: with malfunction of the cerebellum leading to contractions of the extensors with a tendency to move backwards, while the opposite is true of advanced stages of pituitary malfunction, namely contraction of the flexors with resulting forward movements, an antagonistic effect on the direction of movement of these two structures could be explained.

A review of Engel’s thesis, published in the Medizinische Jahrbücher des kaiserl. königl. österr. Staates, (Austrian Annual Medical Journal), reflected that “ *This text is a great testament to the author's honest endeavours and for his talent to work in this field. Even if, over the course of time, the results set out herein, reached through pathological-anatomical examinations and observations, should require modification because of new facts, the methods described and carried out herein as well as the standards of the science still deserve recognition, and in any event, the author has earned lasting credit for sharing such excellent and comprehensive observations*”, a testament any PhD student would still be proud to receive today.

Almost 80 years, two emperors and one unification with Hungary later, the next scientific report of this specimen emerged in the literature in 1904, as part of a large study of pituitary pathology by Jacob Erdheim. In his 200 page paper, “Über Hypophysenganggeschwulste und Hirncholesteatome”, Erdheim defined a cohort of lesions he called “hypophyseal duct tumors” ^4^ (Erdheim 1904). In this paper he describes in detail the pathology of seven such lesions, including that of Josef Ecker. A drawing of the macroscopic pathology of this cases lesion from this paper is shown in Figure 1b. Erdheim hypothesised regarding the existence of two fundamentally different types of pituitary lesions; a “benign” type with a basal papillary morphology and a more aggressive type with histological features resembling those of odontogenic tumours of the jaw, also known as adamantinomas. Following this paper, two of the samples, that described above, and that of a 58-year-old shop keepers widow (Case 2), were stored in Vienna and are currently housed in the Narrentum, part of the Natural History Museum of Vienna.

Over the next century, these tumours were renamed craniopharyngioma, following the influence of the American neurosurgeon Harvey Cushing, who described them as the “most formidable of intracranial tumours” ^5^ (Barkhoudarian and Laws 2013). Craniopharyngiomas are currently classified as either papillary (PCP), predominantly a disease of adults, and adamantinomatous (ACP), the most common tumour of the sellar region in children, based on their histological features and broadly corresponding to the subtypes described by Erdheim ^6^ (Louis, Perry *et al*. 2016). Advances in molecular profiling have now confirmed differing genetic bases to these two subtypes, with PCP usually harbouring V600E mutations in *BRAF* and activating mutations in *CTNNB1* in ACP, and with differing DNA methylation and gene expression profiles ^6^ (Louis, Perry *et al*. 2016).

In a review of Erdheim’s paper in 2015, Case 1, the cystic tumour was re-classified as having features of adamantinomatous pathology ^7^ (Pascual, Rosdolsky *et al*. 2015). In contrast, case 2, showed features of papillary craniopharyngioma. The macroscopic scale of these samples, along with their good state of preservation, anatomically-oriented display and their well characterised clinical histories provided a unique opportunity to study craniopharyngioma using modern techniques ( Figure 1c, Figure 2b).

**Figure 2.**
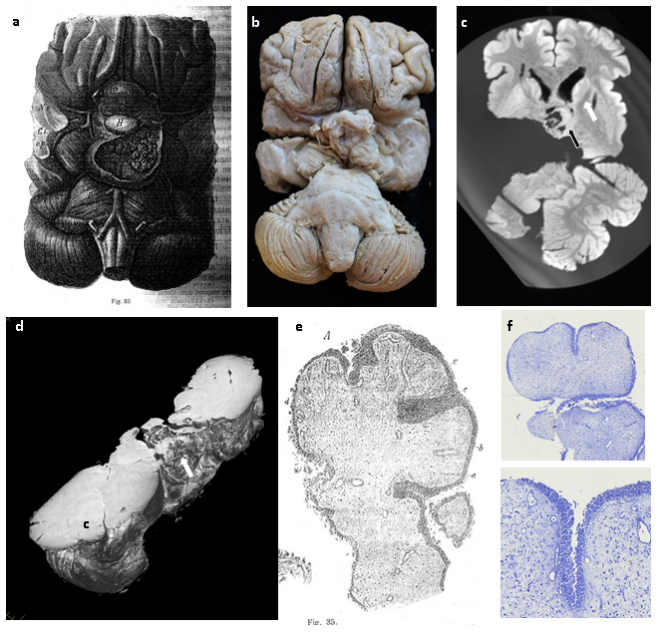
Case 2. **a**) Pathological drawing from Erdheim’s 1904 paper, showing a papillary pituitary growth ^4^. **b**) Macroscopic photograph of the specimen. Note that the specimen has been divided through right temporal lobe. **c**) 3T MRI image showing close relationship of the tumour (black arrow) to the white matter tracts (white arrow). **d**) Micro-CT image showing complex structure and relations of the tumour. C=Cerebellum. **e**) Histological drawing of the tumour by Erdheim. **f**) Toluidine blue staining of a section of the tumour, showing its papillary epithelial nature.

In 2016, we published the first histological scale high resolution 3D imaging of tumour invasion using micro-focus computed tomography (micro-CT). By imaging small pieces of human ACP we were able to visualise, at the cellular scale, the invasion of tumour into surrounding tissue, giving insight into the mechanisms of tumour invasion, and the challenges of achieving complete surgical resection ^8^ (Apps, Hutchinson *et al*. 2016). The samples from these two cases were obtained from Vienna and underwent advanced imaging, initially by 3 Tesla MRI (protocols available on request) then by high resolution micro-CT imaging using a Nikon XT H 225 ST micro-CT scanner, utilising a Molybdenum target to maximise tissue contrast, at 10W scanning power.

Imaging successfully enabled visualisation to a resolution of 61.1μm and 84.6μm respectively for the two cases and facilitated detailed 3D reconstructions of the tumours and the surrounding structures ( Figure 1d–f, Figure 2c,d) (Videos 1–2 ^9, 10^). Case 1, had speckled hyper-intense foci throughout the cyst wall, consistent with calcification, an imaging feature currently used in making supporting a diagnosis of craniopharyngioma. The cyst, while predominantly separated from local structures, was also focally continuous with the surrounding brain, highlighting the challenges in neurosurgical resection ( Figure 1f). In contrast, in case 2, the tumour was more exophytic and heterogeneous in nature. MRI highlighted how the tumour boundary was close to neuronal tracts ( Figure 2c).

**Virtual dissection of 1828 case of craniopharyngioma described by Josef Engel by micro-CT imaging**

*1 Data File*

Video 1: Virtual dissection of case 1 by micro-CT imaging. Tumour cyst, with overlying tentorium cerebelli, brain stem, cerebellum and mid-brain. Bright foci indicate calcification within the cyst wall.

https://dx.doi.org/10.6084/m9.figshare.9724343.v2

**Virtual dissection of 1904 case of craniopharyngioma described by Jacob Erdheim by micro-CT imaging**

*1 Data file*

Video 2: Virtual dissection of case 2 by micro-CT imaging - Tumour with brain stem, cerebellum, midbrain and part of cerebral hemispheres.

https://dx.doi.org/10.6084/m9.figshare.9724703.v3

Tissue sections were also taken for histology. Profiles of Case 1’s cyst showed a surrounding fibrous capsule, and Case 2’s tumour showed cellular architecture consistent with a diagnosis of papillary craniopharyngioma ( Figure 2f). It is likely that the DNA and protein integrity within the tumours suffered due to inadequate fixation over many decades. Although the tumours had been stored in Formalin (Formaldehyde and water) since Erdheim’s study at the start of the 20 ^th^ century, Formaldeyhyde only became routinely used in biological practice during the last decade of the 19 ^th^ century, when Dr Ferdinand Blum discovered its properties as a fixative by accidentally fixing his own fingertips whilst investigating Formaldehyde’s potential antiseptic properties ^3, 11, 12^ (Blum 1893, Fox, Johnson *et al*. 1985, Simmons 2014). Thus, unfortunately, the DNA within the samples was not well preserved and all attempts at sequencing the *CTNNB1* and *BRAF* genes in these cases failed.

In summary, we show two cases which have been investigated over different eras have provided valuable insight into one of the most challenging types of brain tumour. We see how Engel’s interpretation of the function of the pituitary was influenced by the early 19 ^th^ century theories, and Erdheim’s description of hypophyseal duct tumours developed from thorough detailed pathological examination and how combining patients into case series/cohorts facilitates development in medical understanding of diseases Finally we show how modern, advanced imaging techniques can give remarkable detail as to the macro- and micro- anatomy of tumour growth. Such information is valuable for clinicians treating and researching these tumours, in training those in the field and explaining to patients and relatives the challenges involved in managing these tumours.

The ability to perform such analyses on these cases is a testament to the foresight of the founding fathers of modern pathology and the law makers in Vienna in establishing the collection, storage and detailed annotation of specimens for scientific study. Whilst the fundamental scientific methodology of detailed observation remains unchanged, the conclusions of the separate studies highlight how our interpretation of data is shaped by the technologies, methodologies and prevailing theories of the time. How these samples will be interpreted in the next century remains to be seen.

## Ethical approval

This study was performed as part of a larger study, which was approved by a national research ethics committee (REC 13/LO/1494) and all samples handled in accordance with the Human Tissue Act (2004). Routine collection of samples for future analysis was implied under Austrian law at time of collection. 

## Data availability

### Underlying data

No data are associated with this article

### Extended data

Figshare: Virtual dissection of 1828 case of craniopharyngioma described by Josef Engel by micro-CT imaging. https://doi.org/10.6084/m9.figshare.9724343.v2 ^9^

This project contains the following extended data:

- Video 1.mp4 (Video, Virtual dissection of 1828 case of craniopharyngioma described by Josef Engel by micro-CT imaging)
  Figshare: Virtual dissection of 1828 case of craniopharyngioma described by Josef Engel by micro-CT imaging. https://doi.org/10.6084/m9.figshare.9724703.v3 ^10^

This project contains the following extended data:

- Video 2.mp4 (Video, Virtual dissection of 1904 case of craniopharyngioma described by Jacob Erdheim by micro-CT imaging)
  Data are available under the terms of the Creative Commons Attribution 4.0 International license (CC-BY 4.0).
