# Supplementary material for: Learning from cases: Analysis of two cases of craniopharyngioma from the 19 th to the 21 st centuries
Source: F1000Res. 2019 Aug 30;8:1544. [Version 1] doi: 10.12688/f1000research.19626.1 (PMC6993819; doi:10.12688/f1000research.19626.1)
Supplement: Peer review history

# Peer review: Reviewer response for version 1

**DOI:** 10.5256/f1000research.21523.r54329

**Published:** 2020-01-27

**Authors:** Ruth Prieto, Jose M. Pascual

**Affiliations:** 1 Department of Neurosurgery, Puerta de Hierro University Hospital, Madrid, Spain; 2 Department of Neurosurgery, La Princesa University Hospital, Madrid, Spain

This paper by Apps JR *et al*. presents the anatomical, histological and molecular studies of two whole craniopharyngioma-brain specimens from autopsies performed in the 19 ^th^ and 20 ^th^ Centuries. They are preserved at the *Narrenturm, *the site that holds the historical anatomical-pathological collection of Vienna. These tumor specimens, which were instrumental to Viennese pathologist Jakob Erdheim for defining hypophyseal duct tumors or craniopharyngiomas in 1904, are now analyzed with the aid of modern neuroradiological and pathological technologies. Each case corresponds to one of the two major histological craniopharyngioma variants. Case 1 was first macroscopically studied by Joseph Engel, Rokitansky’s disciple and pioneer pathologist, in his original dissertation, “About the Pituitary Gland and the Infundibulum”, published in 1839. This same case was re-examined 65 years later by Jakob Erdheim, who contributed a detailed histological examination of the tumor, which together with 6 additional specimens (including Case 2 in this paper) allowed him to define the new category of hypophyseal duct tumors ( *hypophysenganggeschwülste*). For their part, Apps *et al*. analyzed both cases with 3T-MRI and micro-CT imaging. Nevertheless, they were unable to demonstrate mutations in *β*-catenin/ *CTNNB1 *and *BRAF *genes, the recently identified molecular markers of adamantinomatous and papillary craniopharyngiomas, respectively, due to the poor DNA preservation of the biological tissue of these two old specimens.

 Apps *et al*.’s work supports the importance of historical autopsy specimens, such as these forming the collection of Vienna’s anatomical-pathological museum, as a source of valid scientific information now that new technologies are available to further explore complex pathological entities. Moreover, it demonstrates that old individual specimens from museum collections remain useful even over long periods of time. Apps’s study confirms that Erdheim’s thorough histological studies from the beginning of the 20 ^th^ Century remain completely reliable today. His classification of these two specimens into different craniopharyngioma histological variants, adamantinomatous in Case 1 and squamous-papillary in Case 2, were totally correct. Despite being quite short, the historical background provided by the authors about Engel and Erdheim’s original reports makes reading Apps’s article more pleasant.

 There are some points that the authors may wish to revise to improve their manuscript:

- The word “craniopharyngioma” is misspelled in the Keywords section (there are two “o”).
- It is advisable to use “Joseph” (instead of “Josef”), as this was how Dr. Engel spelled his name in his own articles.
- Authors should consider adding the dates of birth and death of the doctors mentioned throughout the manuscript, Joseph Engel (1816-1899), Carl von Rokitansky (1804-1878), Jakob Erdheim (1874-1937).
- Page 3 (second column): authors mentioned that Engel studied “12 cases of pituitary pathology”, but it is more accurate to say that he studied “12 cases of tumors of the pituitary gland and/or infundibulum”. I recommend reading and discussing the recent article focused on Engel’s dissertation by Pascual JM *et al.* ^1^
- Page 4 (first column, last paragraph): authors mentioned *“Josef Ecker”*, which seems to be an incorrect combination of Dr. Engel’s name and that of his patient (Ecker Johann).
- The videos of the micro-CT scans taken from the two cases do not provide the resolution necessary to study the tumor-brain relationships. Could the authors provide higher-quality images? On the other hand, despite the authors also obtaining the MR imaging of these cases, they only provide one axial image of Case 2. It would be advisable to provide mid-sagittal and coronal-trans-infundibular images of both cases, as these sections are the most useful ones to accurately show the anatomical relationships between these craniopharyngiomas and the adjacent vital neurovascular structures, including the hypothalamus and third ventricle.
- Specific comments for Case 1:
  - It might be useful for readers wishing to study the original articles to clarify that Apps’s Case 1 corresponds to both Case 10 in Engel’s dissertation, which analyzed 12 pituitary/infundibulum tumors, and also to Case 5 in Erdehim’s 1904 article that reviewed 7 tumors.
  - It is inaccurate to describe Case 1 as a *“cystic lesion in the region of the pituitary”* (page 3). It would be better described as a cystic tumor at the base of the brain that had replaced the hypophysis and the infundibulum and extended upwards into the third ventricle and downwards into the sphenoid sinus. 
  - Figure 1a: the position of the figure is wrong; it  should be positioned upside-down (in order to match the tumor views shown in the remaining panels of the figure). In addition, the arrow should point to the same area as the arrow in figure 1b, pointing to an opening at the cyst wall.
  - It would be desirable for the authors to include an image of the histological study performed by Erdheim, as well as their own histological study (as they do in Case 2). On the other hand, since the authors had the chance to take samples of the tumors, if possible, please provide immunostaining for GFAP. On page 5 they stated that the tumor was surrounded by a “fibrous capsule”, but it probably corresponds to reactive gliosis.
- Specific comments for Case 2:
  - As in the previous case, it might be useful to clarify that Case 2 corresponds to Case 6 in Erdehim’s 1904 article.
  - Legend of Figure 2a: it is not correct to describe this tumor as *“a papillar pituitary growth”* as Erdheim’s drawing clearly shows that the pituitary gland (“H”) is intact. This tumor would be better defined as “a papillary infundibular growth”.
  - Figure 2c: I do not understand the reason for the arrow pointing to *“white matter tracts”*. What really matters is the close relationship between the tumor and the hypothalamus, which is the vital structure to consider when planning surgery of these pathological entities.

We confirm that we have read this submission and believe that we have an appropriate level of expertise to confirm that it is of an acceptable scientific standard.

## References

[1] : Joseph Engel (1816–1899), author of a meaningful dissertation on tumors of the pituitary infundibulum: his report on the oldest preserved whole craniopharyngioma specimen. *Virchows Archiv*.2019; 10.1007/s00428-019-02664-z 10.1007/s00428-019-02664-z 31511968

# Peer review: Reviewer response for version 1

**DOI:** 10.5256/f1000research.21523.r53215

**Published:** 2019-09-02

**Authors:** Ashley Grossman

**Affiliations:** 1 Oxford Centre for Diabetes, Endocrinology and Metabolism (OCDEM), University of Oxford, Oxford, UK

This is a brief but fascinating study of the original descriptions of craniopharyngiomas as the tumours were able to be re-imaged using contemporaneous techniques.

 I have no major criticisms, but feel that the figures, which are the dominant feature of this manuscript, could be more sharp, possibly at a higher resolution. In addition, in many cases there are arrows in the figures which are not alluded to in the Legends.

 Finally, a minor point, in the text there is an absence of appropriate apostrophes: Page 3 second column, "This patients's tumour"; Page 4, first column, "shop keeper's widow".

 I also note that the references are given at the end numerically but not in the text.

I confirm that I have read this submission and believe that I have an appropriate level of expertise to confirm that it is of an acceptable scientific standard.
